# Web-based self-help intervention for partners of cancer patients based on acceptance and commitment therapy and self-compassion training: a randomized controlled trial with automated versus personal feedback

**DOI:** 10.1007/s00520-021-06051-w

**Published:** 2021-02-19

**Authors:** Nadine Köhle, Constance H. C. Drossaert, Peter M. ten Klooster, Karlein M. G. Schreurs, Mariët Hagedoorn, Cornelia F. Van Uden-Kraan, Irma M. Verdonck-de Leeuw, Ernst T. Bohlmeijer

**Affiliations:** 1 Department of Psychology, Health & Technology, Centre for eHealth & Well-being Research, University of Twente, P.O. Box 217, 7500 AE, Enschede, The Netherlands; 2 Roessingh Research & Development, P.O. Box 310, 7500 AH Enschede, The Netherlands; 3 Department of Health Sciences, University Medical Center Groningen and University of Groningen, P.O. Box 196, 9700 AD Groningen, The Netherlands; 4 Department of Clinical, Neuro- and Developmental Psychology, Amsterdam Public Health Research Institute, Vrije Universiteit Amsterdam, Amsterdam, The Netherlands; 5 Department of Otolaryngology/Head and Neck Surgery, Amsterdam UMC, location VUmc, P.O. Box 7057, 1007 MB Amsterdam, The Netherlands

**Keywords:** Acceptance and commitment therapy, Cancer, Partners, RCT, Self-compassion, Web-based

## Abstract

**Purpose:**

To evaluate the effectiveness of two versions (personal or automated feedback) of a psychological Web-based self-help intervention targeting partners of cancer patients. The intervention was based on acceptance and commitment therapy (ACT) and self-compassion training. Participants’ adherence and their satisfaction were also studied.

**Methods:**

Two hundred three partners of patients with heterogeneous entities of cancer were randomized into three conditions: personal feedback (PF) (*n* = 67), automated feedback (AF) (*n* = 70), or waiting list (WL) control (*n* = 66). Participants completed measures at baseline (T0) and post-intervention (T1; 3 months after baseline) to assess psychological distress (HADS; primary outcome), positive mental health, caregiver strain, general health (secondary outcomes), posttraumatic growth, resilience, self-compassion, psychological flexibility, sense of mastery, and relational communication style (process measures). Participants in the two experimental conditions also completed these measures at follow-up (T2; 6 months after baseline).

**Results:**

There was no significant difference in change in psychological distress, positive mental health, caregiver strain and general health from T0 to T1 for either of the experimental conditions compared with the WL-condition. However, when compared to a WL-condition, the PF-condition was effective in increasing psychological flexibility (effect size *d* = 0.49) and resilience (*d* = 0.12) and decreasing overprotection (*d* = 0.25), and the AF-condition was effective in reducing overprotection (*d* = 0.36) and improving protective buffering (*d* = 0.36). At follow-up, the PF-condition was more effective than the AF-condition for improving mental health (*d* = 0.36), psychological flexibility (*d* = 0.60), mastery (*d* = 0.48), and protective buffering (*d* = 0.24). Participants positively appreciated the intervention and 69% participants were adherent.

**Conclusion:**

This study demonstrates that a Web-based intervention based on ACT and self-compassion training with automated or personal feedback does not seem to improve psychological distress; however, it may have the potential to support partners of cancer patients to cope with the difficult situation they are facing. The condition with personal feedback seemed to be more beneficial.

## Background

Cancer affects not only the lives of the patients but also those of their partners or spouses [1, 2]. For many of such caregivers, it is difficult to disengage themselves from their caregiving situation, as they long to help the patient to feel better or feel a sense of duty to support the patient in every possible way [3, 4]. Beyond these highly demanding responsibilities, partners also have to maintain their regular activities such as household tasks, child care, and work responsibilities [4]. This challenging situation often leads to increased psychological distress (e.g., [5]), increased caregiver strain, deteriorated physical health, and diminished social and relationship functioning (e.g., [4, 6, 7]).

To help partners maintain their own health and, in turn, provide the best possible care for the patient, evidence-based and easily accessible interventions for partners are needed. Although some of these interventions have become available during the last decade, most partners do not use these interventions because they are not aware of their own health problems, they have a lack of time, or they are reluctant to seek help [2, 3, 8, 9]. Web-based interventions can surmount these barriers, as they would inherently allow partners to receive help at home at any convenient time and, if they prefer, to remain anonymous [10–12]. Research has shown that cancer caregivers frequently use the Internet to receive information and support [2, 13], and that they are receptive to Web-based tools that might help with their caregiving tasks and reduce their caregiver strain [2, 3, 11]. However, the Internet has rarely been used to deliver psychological interventions to partners of cancer patients [10, 12]. Therefore, we developed a theory-driven, Web-based self-help intervention called *Hold on, for each other* which aims “to help partners to positively persevere during the difficult times they find themselves facing” [14]. To ensure that the intervention complied with partners’ needs, we actively involved them in the development process of this new intervention [3, 15] (for a detailed description of the development process, please see Köhle et al. [16]).

The intervention is based on acceptance and commitment therapy (ACT) [17] and self-compassion training. ACT aims to increase people’s psychological flexibility, which can be defined as “the ability to contact the present moment more fully as a conscious human being, and to change or persist in behaviour when doing so serves valued ends” [18]. ACT tries to help people to accept unavoidable aspects in life, be mindfully present, choose important values in life and live in accordance to those values [17]. To our knowledge, ACT has not been applied to the context of partners of cancer patients yet, despite the potential benefits it can have for this group. Research has shown that partners are often confronted with unhelpful thoughts and negative feelings (e.g., [4]). Consequently, ACT could help them to disengage from these unhelpful thoughts and feelings, learn them to engage more fully in the present moment instead of getting lost in “what-if” scenarios, and bring them closer to their personal values. Self-compassion is the ability to act with kindness and understanding towards yourself whenever you are having a difficult time [19]. Self-compassion may be particularly relevant to partners since they usually have high expectations of themselves; many would do anything to help the patient feel better, regardless of the personal cost or consequences. While engaged in this highly demanding act, most partners frequently neglect their own health problems, overlook their personal needs, and even feel guilty when they do eventually meet their own needs or enjoy pleasurable things [20].

Feedback is an essential component of Web-based interventions because it can improve adherence (e.g., [21]). We chose to examine two versions of feedback (personal versus automated feedback) because our previous studies indicated that partners have different needs regarding feedback [3, 15]. Also, research showed that an automated feedback Web-based intervention for people with mild depressive symptoms can be as effective and reach similar adherence as the same intervention with personal feedback [22]. This is interesting because automated feedback could increase cost-effective upscaling.

This study aimed to evaluate the effectiveness of two versions of *Hold on, for each other* on partners’ psychological distress, positive mental health, caregiver strain, general health, psychological flexibility, self-compassion, resilience, posttraumatic growth, sense of mastery, and relational communication style. Participants’ adherence and their satisfaction with the intervention were also studied.

## Methods

CONSORT guidelines for randomized trials were followed. The protocol of this study is described in detail by Köhle et al. [16]. We carried out a prospective randomized controlled trial with three conditions: a waiting list control condition (WL-condition), a personal feedback condition (PF-condition), and an automated feedback condition (AF-condition) (allocation ratio 1:1:1). Randomization was stratified for gender and self-reported stage of disease and was conducted a priori using a computer-generated random sequence of numbers, made with randomizer.org. This sequence was concealed and only used when the next participant had to be assigned. The first author was responsible for randomization, enrolment, and the assignment of participants. Participants received the outcome of the randomization via e-mail, and people from the two experimental conditions received a link to start the intervention. Participants were aware of the different conditions, and they started the intervention at an individual time point (directly after randomization).

All participants completed Web-based questionnaires at baseline (T0) and post-intervention (T1; 3 months after baseline). Participants in the two experimental conditions also completed these measures at follow-up (T2; 6 months after baseline). For ethical considerations, we chose to offer participants in the WL-condition access to the intervention with automated feedback after they completed the T1-measurement. Before the start of the intervention, participants in the WL-condition were free to access other forms of care. This study was approved by the Twente Medical Ethics Committee under the file number P13-17 (Dutch trial register: NTR4035).

### Description of the intervention

The intervention consists of six modules (plus two optional modules), which can be worked through in 6 to 12 weeks. Participants were asked for a minimum time investment of 1 to 1.5 h per week. Each module includes psychoeducation, psychological and meditation exercises, tips, references to relevant websites, inspiring texts/poems, and an (optional) weekly text message service with short inspiring texts. In our needs assessment [3], we found that partners had a need for some form of peer support; however, some were afraid of being confronted with negative stories. Therefore, we embedded low-threshold, positive peer support options: participants could share answers on exercises with other participants, they could exchange tips, or they could contact other participants in private e-mail conversations. Participants could stay anonymous. An overview of the focus of each module, underlying theories, and example exercises can be found in Köhle et al. [14].

Participants in the PF-condition received feedback in a form of weekly e-mail messages from a personal counsellor at a fixed day of the week. After completing a module, these participants were encouraged to e-mail their counsellor about their experiences with the module. Subsequently, the counsellor replied with a reflection on the participant’s progress in the module, and feedback on key exercises. Counsellors were five trained master psychology students. During their training, they received information on challenges partners of cancer patients are confronted with, development process of the intervention, theoretical background, study design and aims, and writing e-mails to the participants in a structured way (compliment participant about progress, provide a review of past module including feedback on key exercises and lessons learned, answer participant’s questions/reactions and problems, preview the upcoming topic(s), and motivate participant to proceed). All communication was provided within the enclosed and encrypted intervention’s Web-based system.

Participants in the AF-condition received short, pre-programmed feedback messages (appearing in a pop-up window) immediately after completing key exercises of the module. These messages consisted of generic and more reflective content with the aim to normalize and validate emotions and reactions participants could experience after doing an exercise. The content was developed based on theoretical insights concerning ACT and/or self-compassion and experiences we have with these kinds of exercises. An example of such a feedback message is “It often seems easier to be friendly to others than to ourselves. Maybe you could try to treat yourself like a good friend”. The content of these messages was the same as the feedback on the key exercises that participants in the PF-condition received.

### Participants

Participants were recruited from February 2014 to June 2015 through a multi-component recruitment approach (e.g., via national newspapers, patient organizations, hospitals, and psycho-oncological centres) throughout The Netherlands. Applicants were referred to a website where they could find information about the study and the intervention and where they could apply for participation. Inclusion criteria were > 18 years, being a partner of a cancer patient/survivor, having Internet access, mastery of Dutch, and having a score of >3 on the Hospital Anxiety and Depression Scale (HADS) [23]. The relatively low score on the HADS was chosen, because we wanted to offer a low-threshold intervention for partners who felt themselves in need for psychological help, and yet leave some room for improvement. Exclusion criteria were anxiety and/or depressive symptoms (self-reported during the application process or a score ≥ 15 on HADS anxiety and/or depression), having recently (< 3 months ago) started with psychopharmacological treatment, currently receiving psychological treatment, not being able to spend 1–1.5 h on the intervention, cancer diagnosis of the patient was < 3 months ago, and patient had died.

### Power analysis

Sample size was calculated conservatively based on the ability to detect at least an effect size of 0.50 (Cohen’s *d*) in the post hoc tests on the primary outcome at T1 and follow-up T2 with a power of (1-beta) = 0.80 in a two-tailed test (*p* < .05). According to this analysis, 64 participants per condition were needed. The sample size was increased by another five participants per condition to consider non-normal distribution and possible post hoc analyses. Anticipating a drop-out rate of 20% between T0 and long-term follow-up (T3; not included in this study), our a priori goal was to include 87 participants in each condition at baseline.

### Measures

#### Primary outcome

Psychological distress was assessed with the HADS (14 items; range 0–42) [23]. Higher scores on this scale indicate more psychological distress.

#### Secondary outcome

Positive mental health was measured with the Mental Health Continuum Short-Form (MHC-SF; 14 items; range 1–6) [24]. Higher mean scores indicate higher levels of mental health. Caregiver strain was measured with the Caregiver Strain Index (CSI; 13 items; range 0–13) [25]. Higher scores suggest that caregivers experience more strain due to their caregiving tasks. General health was measured with one item of the RAND 36 (range 1–5; higher scores indicating better general health) [26, 27].

#### Process

Psychological flexibility and self-compassion were added because they are supposed to be influenced by the theoretical approaches ACT and self-compassion [4, 17, 20]. Furthermore, resilience, posttraumatic growth, sense of mastery, and different styles of relational communication were chosen because they were derived from previous studies (e.g., [1, 4, 28]) examining the effects of cancer on the partner’s life and relationships. Psychological flexibility was measured with the Acceptance and Action Questionnaire II (AAQ-II; 7 items; range 7–49) [29]. Higher scores indicate higher levels of psychological flexibility. Self-compassion was assessed with the Self-Compassion Scale Short-Form (SCS-SF; 12 items; mean range 1–7) [30]. Higher mean scores on the SCS-SF indicate that individuals are more self-compassionate. Resilience—the ability to bounce back or recover from stress—was measured with the Brief Resilience Scale (BRS; 6 items) [31]. The mean score can range from 1 to 5, with higher scores indicating being more resilient. Posttraumatic growth—referring to positive experiences as a result of a traumatic event—was measured with the Posttraumatic Growth Inventory Short-Form (PTGI-SF; 10 items; range 0–50) [32]. Higher scores suggest more posttraumatic growth. Sense of mastery was measured with the Pearlin Mastery Scale (PMS; 5 items; range 5–25) [33], with higher scores signifying that the individual perceives more control over his/her life. The different relational communication styles were assessed with the Active Engagement Scale [34]. This scale measures (1) active engagement (i.e., involving the patient in discussions; 5 items), (2) protective buffering (i.e., hiding one’s concerns; 8 items), and (3) overprotection (i.e., underestimation of the patient’s capabilities, resulting in unnecessary help; 6 items) [35]. For each subscale, a mean score was calculated, with higher scores indicating higher levels of active engagement, protective buffering, and overprotection (range 1–5).

#### Satisfaction, adherence, demographics, and clinical characteristics

Satisfaction with the intervention was measured with the Client Satisfaction Questionnaire (CSQ-8; 8 items; range 1 (very negative) to 4 (very positive)) [36]. Participants also rated the intervention on a scale from 1 (extremely poor) to 10 (excellent). Adherence to the Web-based intervention was obtained via log files (log-in to modules yes/no). Participants who reached module 6 were classified as adherent.

A study-specific questionnaire was used to obtain partners’ personal characteristics and patients’ cancer-related characteristics.

### Statistical analysis

Analyses were performed using SPSS 24.0. All tests were two-tailed. One-way analysis of variance (ANOVA) and *χ*^2^ tests were conducted to examine baseline differences between the conditions, on any of the socio-demographics, cancer-related characteristics, and outcome measures. Little’s MCAR test indicated that missing data were completely at random (*χ*^2^ (530) = 385.47, *p* = 1.000). An intention-to-treat (ITT) analysis and per-protocol (PP) sensitivity analysis of completers only were conducted using the linear mixed model (LMM) procedure.

Mean scores of the primary and secondary outcome measures on T0 and T1 (PF- versus WL-condition and AF- versus WL-condition) and on T0, T1, and T2 (PF- versus AF-condition) were analysed using LMM, with time as repeated measure and group, time, and group × time interaction entered as fixed effects. The estimation method used was restricted maximum likelihood (REML) in all models and the covariance type was specified as compound symmetry as this structure was the best fit for most outcome measures. In case of a significant condition-by-time interaction between the AF- and PF-conditions, an additional ANCOVA, based on observed data, with T0 as covariate was conducted to test the differences in PF- versus AF-condition on T1 and T2. Between-group standardized effect sizes (ES) at T1 and T2 were calculated based on the estimated marginal means and corresponding standard errors from the LMMs. ES were expressed as Cohen’s *d* (Δ estimated marginal mean / pooled standard deviation). ES of 0.20 were considered small, 0.50 moderate, and 0.80 large [37].

We evaluated participants’ adherence, satisfaction, and drop-out with the use of descriptive statistics, *χ*^2^ tests, and one-way ANOVAs.

## Results

### Study population

**Fig. 1:**
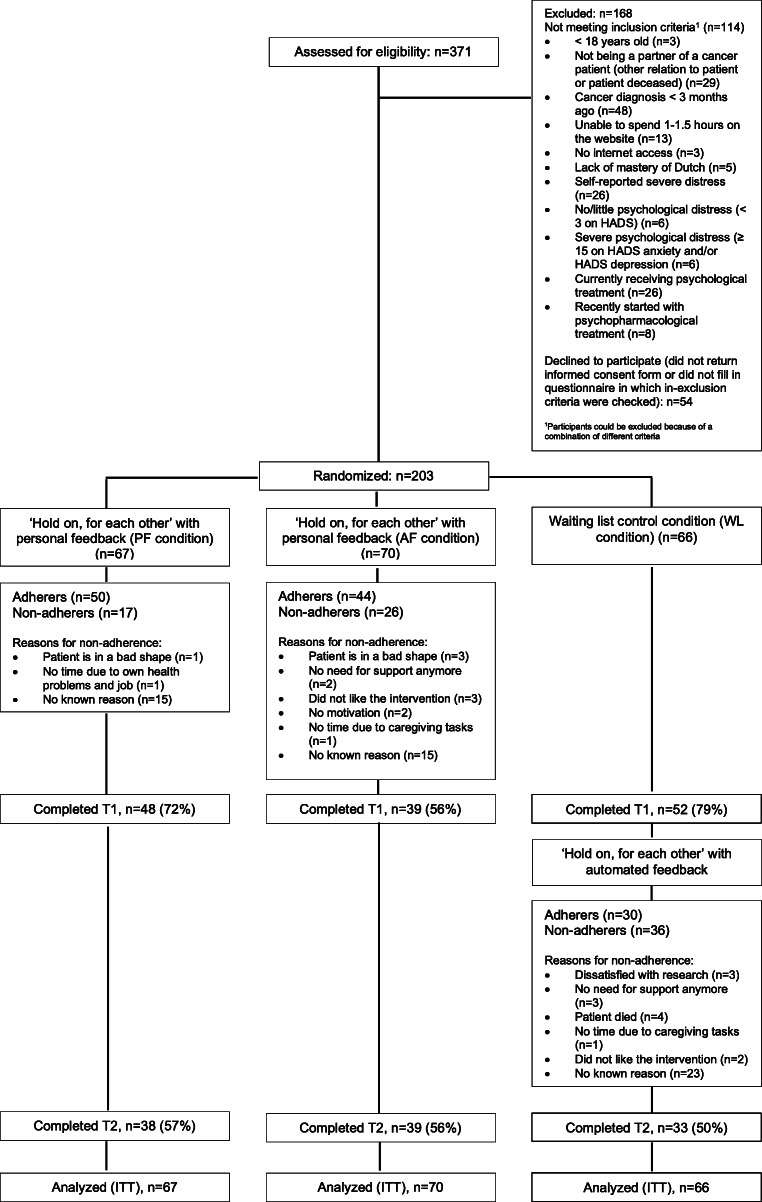
Flow chart of the study. T1, post-intervention (3 months after baseline); T2, follow-up (6 months after baseline)

Three hundred seventy-one partners expressed an interest to participate in the intervention. Based on the in- and exclusion criteria, 114 applicants were excluded before randomization. Of all applicants, 54 did not return the informed consent form, or did not fill in the questionnaire in which the in- and exclusion criteria were checked. The remaining 203 participants were randomized to one of the three conditions after receiving their informed consent and completing the baseline questionnaire (T0) (Fig. 1). Person- and cancer-related characteristics appear in Table 1. ANOVA and *χ*^2^ tests showed that there were no significant differences at baseline between randomized conditions for any of the person- and cancer-related characteristics and outcome measures.

**Table 1:** Partners’ personal characteristics and patients’ cancer-related characteristics

	Total (*n* = 203)		PF (*n* = 67)		AF (*n* = 70)		WL (*n* = 66)		*p*^b^
	*n*	%	*n*	%	*n*	%	*n*	%	
Partners’ characteristics									
Gender (*n* = 203)									.974
Female	143	70.4	47	70.1	50	71.4	46	69.7	
Male	60	29.6	20	29.9	20	28.6	20	30.3	
Age, mean (SD), years [range] (*n* = 203)	55.89 (10.72) [27–82]		56.97 (9.88) [29–82]		56.40 (11.15) [30–79]		54.24 (11.03) [27–79]		.251
Country of birth (*n* = 203)									.177
The Netherlands	193	95.1	61	91.0	68	97.1	64	97.0	
Other	10	4.9	6	9.0	2	2.9	2	3.0	
Children (*n* = 203)									.240
No/or living away from home	131	64.5	43	64.2	50	71.4	38	57.6	
Yes, living at home	72	35.5	24	35.8	20	28.6	28	42.4	
Education (*n* = 197)									.065
Low	32	16.2	6	9.0	14	20.0	12	18.2	
Middle	58	29.4	19	28.4	25	35.7	14	21.2	
High	107	54.3	41	61.2	28	40.0	38	57.6	
Employment (*n* = 203)									.184
Paid job	121	59.6	41	61.2	36	51.4	44	66.7	
No job	82	40.4	26	38.8	34	48.6	22	33.3	
Patients’ cancer-related characteristics									
Sort of cancer (*n* = 199)									.254
Colon cancer	28	14.1	8	11.9	7	10	13	19.7	
Prostate cancer	24	12.1	5	7.5	12	17.1	7	10.6	
Lung cancer	23	11.6	5	7.5	9	12.9	9	13.6	
Breast cancer	18	9.0	8	11.9	8	11.4	2	3.0	
Lymph node cancer	17	8.5	9	13.4	5	7.1	3	4.5	
Head and neck cancer	11	5.5	5	7.5	3	4.3	3	4.5	
Leukemia	11	5.5	6	9.0	1	1.4	4	6.1	
Bone marrow cancer	11	5.5	4	6.0	3	4.3	4	6.1	
Brain tumor	8	4.0	1	1.5	4	5.7	3	4.5	
Kidney cancer	8	4.0	1	1.5	4	5.7	3	4.5	
Other^a^	40	20.1	15	22.4	13	18.6	12	18.2	
Time since diagnosis (*n* = 203)									.600
Between 3 and 6 months ago	43	21.2	13	19.4	12	17.1	18	27.3	
Between 6 and 12 months ago	47	23.2	15	22.4	18	25.7	14	21.2	
1–5 years ago	81	39.9	28	41.8	27	38.6	26	39.4	
5–10 years ago	19	9.4	6	9.0	10	14.3	3	4.5	
> 10 years ago	13	6.4	5	7.5	3	4.3	5	7.6	
Current treatment (*n* = 201)									.982
No	78	38.8	26	38.8	27	38.6	25	37.9	
Yes	123	61.2							
Self-reported phase of disease (*n* = 203)									.995
Patient is still in treatment with curative intent	52	25.6	17	25.4	18	25.7	17	25.8	
Treatment with curative intent is completed; patient is recovered	33	16.3	12	17.9	11	27.5	10	15.2	
Patient will (probably) not recover	118	58.1	38	56.7	41	58.6	39	59.1	

*PF*, personal feedback; *AF*, automatic feedback; *WL*, waiting list

^a^17 different sorts of cancer

^b^One-way ANOVA for age, chi-square tests for the remaining variables

### Treatment adherence and drop-out

Overall, 69% completed the intervention (75% in PF- versus 63% in AF-condition (*χ*^2^ = 2.2, *p* = .138)). Reasons for non-adherence appear in Fig. 1. Adherers and non-adherers did not differ significantly on person- and cancer-related characteristics and outcome measures at baseline.

Participants in all conditions (*n* = 87) reported to have spent on average 108 min per week on the intervention (PF-condition 120 min versus AF-condition 92 min (*F* = 2.28, *p* = .135)). At T1, data were available for 139 participants (drop-out rate 32%), and at T2 for 110 participants (drop-out rate 46%; see Fig. 1). Neither significant baseline differences on any of the outcome measures existed between participants who did or did not complete all questionnaires (T0, T1, T2) nor were there significant baseline differences between participants who did or did not complete T1 and T2. Data regarding treatment adherence and drop-out are not shown in table.

### Outcomes

LMM (Table 2) revealed no significant interaction effects from T0 to T1 for the primary and secondary outcomes for either of the intervention conditions compared with the WL-condition. However, a significant difference between the PF- and WL-conditions was found for psychological flexibility (*p* = .042), resilience (*p* = .023), and overprotection (*p* = .028) at T1, with effect sizes of .49, .12, and .25 respectively in favour of PF compared to the WL-condition. Also, a significant difference between AF- and WL-conditions was found at T1 for protective buffering (*p* = .034) and overprotection (*p* = .004), with effect sizes of .36 for both outcomes in favour of the AF-condition. Per-protocol analyses (data not displayed in paper) showed a significant interaction effect on psychological distress (primary outcome) for PF- versus WL-condition (*F* = 5.99, *p* = .017) and AF- versus WL-condition (*F* = 5.41, *p* = .023), with effect sizes of .38 and .41 respectively both in favour of the intervention conditions. Moreover, when looking at the secondary and process measures, PP analyses showed overall similar patterns as in the ITT analyses. Yet, we found additional significant interaction effects on resilience (AF- versus WL-condition: *F* = 4.36, *p* = .041) and on protective buffering (PF- versus WL-condition: *F* = 6.60, *p* = .012), with effect sizes of .32 and .31 respectively in the expected directions.

**Table 2:** Outcome measures at baseline, post-intervention, and follow-up and results of mixed model analysis

Outcome								Condition-by-time interaction, *F* (*p*), T0-T1		Condition-by-time interaction, *F* (*p*), T0-T1-T2
	Time	PF (*n* = 67)		AF (*n* = 70)		WL (*n* = 66)		PF-WL	AF-WL	PF-AF
		*M*	SE	*M*	SE	*M*	SE			
Primary outcome measure										
Psychological distress ↓	T0	12.5	0.7	12.4	0.7	12.7	0.7	2.87 (.093)	2.98 (.087)	1.21 (.301)
	T1	12.4	0.8	12.8	0.9	14.7	0.8			
	T2	11.8	0.9	13.5	0.9	-	-			
Secondary outcome measure										
Positive mental health ↑	T0	4.2	0.1	4.2	0.1	4.1	0.1	1.54 (.218)	.12 (.736)	3.26 (.041)*
	T1	4.2	0.1	3.9	0.1	3.9	0.1			
	T2	4.1	0.1	3.8	0.1	-	-			
Caregiver strain ↓	T0	7.5	0.3	6.9	0.3	7.3	0.3	1.51 (.223)	.01 (.913)	1.47 (.223)
	T1	6.9	0.4	6.8	0.4	7.3	0.3			
	T2	6.5	0.4	6.9	0.4	-	-			
General health ↓	T0	3.0	0.1	3.0	0.1	3.0	0.9	.68 (.413)	.03 (.869)	.66 (.516)
	T1	3.0	0.1	3.1	0.1	3.1	0.1			
	T2	2.9	0.1	3.1	0.1	-	-			
Process measures										
Psychological flexibility ↑	T0	5.2	0.1	5.4	0.1	5.2	0.1	4.26 (.042)*	2.07 (.154)	7.57 (.001)***
	T1	5.3	0.1	5.4	0.1	4.9	0.1			
	T2	5.5	0.1	5.0	0.1	-				
Self-compassion ↑	T0	4.5	0.1	4.7	0.1	4.6	0.1	2.22 (.139)	2.48 (.119)	3.16 (.045)*
	T1	4.6	0.1	4.8	0.1	4.5	0.1			
	T2	4.7	0.1	4.5	0.1	-	-			
Resilience ↑	T0	3.1	0.1	3.2	0.1	3.2	0.1	5.36 (.023)*	3.16 (.078)	.16 (.854)
	T1	3.3	0.1	3.3	0.1	3.2	0.1			
	T2	3.1	0.1	3.2	0.1	-	-			
Posttraumatic growth ↑	T0	9.6	0.9	8.9	0.9	8.9	0.9	.72 (.399)	.18 (.675)	.95 (.388)
	T1	16.2	1.0	14.0	1.0	14.6	0.9			
	T2	16.7	1.1	14.7	1.0	-	-			
Sense of mastery ↑	T0	3.3	0.2	3.3	0.1	3.1	0.1	.13 (.723)	.08 (.775)	4.65 (.011)*
	T1	3.4	0.1	3.4	0.1	3.2	0.1			
	T2	3.5	0.1	3.1	0.1	-				
Active engagement ↑	T0	4.3	0.1	4.3	0.1	4.2	0.1	.66 (.419)	.10 (.752)	.17 (.831)
	T1	4.2	0.1	4.1	0.1	4.1	0.1			
	T2	4.1	0.1	4.1	0.1	-				
Protective buffering ↓	T0	2.4	0.1	2.4	0.1	2.4	0.1	3.06 (.083)	4.63 (.034)*	4.48 (.013)*
	T1	2.3	0.1	2.2	0.1	2.5	0.1			
	T2	2.2	0.1	2.4	0.1	-				
Overprotection ↓	T0	2.2	0.1	2.2	0.1	2.1	0.1	4.95 (.028)*	8.93 (.004)**	.11 (.895)
	T1	2.1	0.1	2.0	0.1	2.3	0.1			
	T2	2.1	0.1	2.1	0.1	-				

*PF*, personal feedback; *AF*, automatic feedback; *WL*, waiting list

*M*, estimated marginal mean; *SE*, standard error

Arrows (↑ or ↓) indicate the desirable direction for each of the outcome measures

*p < .05; **p < .01; ***p < .001 (two-tailed)

Since participants in the WL-condition started the intervention after they completed T1, we did not have their comparison data for T2. However, we examined the differences between the PF- and AF-conditions on T1 and T2. There was no significant condition-by-time effect (*p* = .301) with respect to psychological distress. However, we found significant interaction effects for partners’ positive mental health (*p* = .041), psychological flexibility (*p* = .001), self-compassion (*p* = .045), sense of mastery (*p* = .011), and protective buffering (*p* = .013) from T0 to T2. ANCOVAs (Table 3) revealed no significant differences between the experimental conditions at T1. At T2, however, significant differences were found for mental health (*p* = .031), psychological flexibility (*p* = .008), sense of mastery (*p* = .005), and protective buffering (*p* = .028), with effect sizes (.36, .60, .48, and .24, respectively) in favour of the PF-condition. PP analyses (data not displayed in the paper) showed similar patterns as in the ITT analyses. There was no significant condition-by-time effect with respect to psychological distress (*F* = .244, *p* = .784), but we found significant interaction effects for partner’s psychological flexibility (*F* = 4.49, *p* = .013) and sense of mastery (*F* = 3.19, *p* = .044) from T0 to T2. ANCOVAs only revealed significant differences between PF and AF at T2 for both psychological flexibility (*F* = 4.41, *p* = .040) and sense of mastery (*F* = 5.51, *p* = .022), with effect sizes (.29 and .58 respectively) in favour for the PF-condition.

**Table 3:** Results of ANCOVAs to examine differences in the two intervention groups at T1 and T2

Outcome	T1 (*n* = 87)	T2 (*n* = 77)
	*F* (*p*)	*F* (*p*)
Positive mental health	2.14 (.147)	4.86 (.031*)
Psychological flexibility	.31 (.577)	.742 (.008**)
Self-compassion	.40 (.531)	2.91 (.092)
Sense of mastery	.11 (.746)	8.21 (.005**)
Protective buffering	.38 (.538)	5.04 (.028*)

*p < .05; **p < .01; ***p < .001 (two-tailed)

### Satisfaction

Participants were generally satisfied with the intervention (average score = 3.0; SD = 0.6 on the CSQ-8). Most participants rated the quality of the intervention as good. In total, 84.0% would recommend it to other people in need of similar help, and 81.0% indicated that they received the kind of support they wanted. On a scale from 1 to 10, the intervention was evaluated with a 7.4 (SD = 1.3, *n* = 87). There were neither significant differences in the rating by the participants in the PF- versus AF-conditions (CSQ-8: *F* = .722, *p* = .398; grade: *F* = .149, *p* = .701), nor were there significant differences concerning self-reported stage of disease (CSQ-8: *F* = .001, *p* = .999; grade: *F* = .342, *p* = .712).

## Discussion

This intervention has the potential to support partners of cancer patients. While there were no significant effects on primary and secondary outcomes, we did find improvements in several of the process measures. In addition, PP sensitivity analyses suggested that the intervention was effective in reducing psychological distress for those participants who were adherent to the intervention. The PF-condition seemed to be more beneficial than the AF-condition. Partners in the PF-condition experienced more psychological flexibility and resilience at T1, whereas these variables decreased or stayed the same in partners in the WL-condition. Furthermore, partners in the PF-condition improved regarding overprotection, whereas this variable deteriorated in the WL-condition from T0 to T1. In the AF-condition, partners only improved regarding protective buffering and overprotection in comparison with the WL-condition.

In the ITT analyses, we also found significant differences between the PF- and AF-conditions in positive mental health, psychological flexibility, sense of mastery, and protective buffering (and in the PP sensitivity analyses for psychological flexibility and sense of mastery) at T2 in favour of the PF-condition. One explanation for this finding could be that the personal feedback fits the partners’ needs better than the automated feedback. Another explanation could be that the weekly communication with the counsellor may have been more motivating, resulting in more commitment to the intervention. Participants in the PF-condition received slightly longer feedback messages and spent more (albeit not significantly) time on the website, and more participants in this condition completed the intervention. In addition, sharing experiences with the counsellor and actively reflecting on the content of the intervention might have been a key process in finding recognition and helping participants to better cope with their situation, internalize lessons learned, and then apply them in their daily life.

Our findings contradict previous research in which a Web-based intervention with automated feedback for people with depression and anxiety was as effective as the same intervention with personal feedback [22]. However, this apparent contradiction can be explained by the different forms of feedback. One difference with the previous study is that they used nearly the same set-up (considering length and presentation) of the human and automated feedback. Both groups received weekly feedback messages, whereas our PF-condition received weekly messages and the AF-condition received the feedback directly after completing key exercises. It is possible that our AF-participants processed the feedback less profoundly than participants who received the feedback delayed. To our knowledge, Kelders et al. [22] were the first to compare a Web-based intervention with two versions of feedback. Evidently, more research into automated feedback for Web-based interventions is needed. Moreover, the effectiveness of the modality of delivering (personal versus automated feedback) may also differ between users. Future studies should reveal if the concordance between a person’s preference and the actual modality contributes to the appreciation and effectiveness of the intervention.

The small to moderate effect sizes between the conditions at post-intervention and follow-up are in line with other (Web-based) caregiver interventions [10, 38]. The relatively small effect sizes between the two intervention conditions were expected since the conditions only varied regarding the form of the provided feedback. The question remains whether it would be worth the effort and extra costs to add more personal feedback to the intervention. More research into the cost-effectiveness is recommended.

Despite the fact that there was no significant change in psychological distress, partners were highly satisfied with the intervention, which is in line with the results of our qualitative evaluation study in a subsample of participants [14]. The positive evaluation and the relatively high adherence rates may be the result of our participatory developmental process, in which we actively involved the partners in all phases [16].

Nevertheless, we did have difficulties with inclusion and high drop-out rates. Maybe we failed to detect differences between the conditions because our study lacked power. Notably, 31% of the applicants were excluded from our study, mainly because the patient’s cancer diagnosis was less than 3 months ago, applicants were not the partner of the cancer patient (but a child, parent, or other family caregiver), the patient was deceased, the applicant reported suffering from severe distress, or they were currently receiving psychological treatment. Future studies are needed to examine if the current intervention can be adapted to the excluded applicants.

## Study limitations

This study has five main limitations. First, as stated above, our study was slightly underpowered. Second, partners’ baseline distress was relatively low when compared to the results of previous studies (e.g., [39]), leaving less room for improvement in the primary outcome [40]. Third, we could not test whether the improvements of the PF- and AF-conditions from T1 to T2 were significant compared with the WL-condition, since the participants in the WL-condition could access the intervention with automated feedback after they completed T1. Fourth, because we deemed the intervention as less suitable, we excluded partners with severe distress. Instead, we encouraged them to seek professional help immediately. We also excluded partners of cancer patients whose partner’s cancer diagnosis was < 3 months ago because we thought that in this difficult period, there might be too little time and energy available to spend on the intervention. Possibly, a blended form of this intervention could adequately support these groups as they could use the intervention under professional guidance. Yet, more research into this option is needed. Finally, considering the relatively small sample size and the explorative character of the secondary and process outcome analyses, we did not correct our *p*-values for multiple testing. Therefore, significant outcomes need to be interpreted with caution.

## Conclusions

This intervention based on ACT and self-compassion training with automated or personal feedback does not seem to improve psychological distress, but it may have the potential to support partners of cancer patients to cope with the difficult situation they are facing. The personal feedback condition seems to be more beneficial.

## Data Availability

Datasets are available from the corresponding author on reasonable request.
